# Endocrine Phenotype and GH1 Sequence Variation in Children with Short Stature: Five-Year Growth Outcomes of Growth Hormone Therapy

**DOI:** 10.3390/genes17101279

**Published:** 2026-10-14

**Authors:** Serpil Albayrak, Murat Karaoglan, Ahmet Yildirim, Mehmet Keskin

**Affiliations:** 1 Department of Pediatric Endocrinology, Gaziantep Cengiz Gökçek Maternity and Children’s Hospital, 27010 Gaziantep, Türkiye; 2 Department of Pediatric Endocrinology, Faculty of Medicine, Gaziantep University, 27080 Gaziantep, Türkiye; muratkaraoglan@hotmail.com (M.K.); ahmet161720@gmail.com (A.Y.); mkeskin@gantep.edu.tr (M.K.)

**Keywords:** idiopathic short stature, growth hormone deficiency, growth hormone therapy, genetic variants, longitudinal growth

## Abstract

**Background:** Growth hormone (GH) stimulation tests have limited diagnostic precision in childhood short stature (SS), and the relationships among endocrine phenotype, GH1 variation, and long-term treatment response remain unclear. We examined the clinical, endocrine, genetic, and five-year growth outcomes of children receiving recombinant GH. **Methods:** In this retrospective cohort study, 74 children with SS were classified as idiopathic SS (ISS; *n* = 28) or growth hormone deficiency (GHD; *n* = 46) using available GH stimulation-test results together with the integrated clinical, auxological, biochemical, radiological, and treatment-eligibility assessments documented in the medical records. **Results:** Median annual height velocity was 9.0 cm/year, median height gain was 1.80 standard deviation scores (SDS), and median IGF-1 increased from 125.5 to 306 µg/L. GH dose, endocrine parameters, annual height velocity, height gain SDS, and mean post-treatment height and weight gain did not differ significantly between groups. Height and weight increased over follow-up (both *p* < 0.001), with no significant group or time-by-group interaction. *GH1* sequencing predominantly identified common promoter/upstream and intronic polymorphisms; no pathogenic or likely pathogenic variants were detected. Exploratory genetic clustering showed no clinically meaningful association with endocrine phenotype or treatment response. **Conclusions:** Adjusted models detected no statistically significant between-group differences in height or weight trajectories. This finding does not establish equivalent treatment efficacy.

## 1. Introduction

Short stature (SS) is among the most common reasons for referral to pediatric endocrinology and affects roughly 2–3% of children. It is defined as a height below −2 standard deviation score (SDS) for age and sex, and it spans a wide range of endocrine, genetic, nutritional, chronic systemic, skeletal, and constitutional causes [1,2]. Even after a thorough work-up, close to half of these children have no definitive diagnosis and are labeled as having idiopathic SS (ISS), a reminder of how many biological and environmental factors converge on linear growth [2,3].

The standard evaluation of SS combines auxology, growth velocity, bone age, serum insulin-like growth factor-1 (IGF-1), pituitary magnetic resonance imaging (MRI), and GH stimulation testing [4]. GH stimulation tests, however, are poorly reproducible, vary with method, and give values that overlap between ISS and partial GH deficiency (GHD). Current guidance therefore treats the stimulation result as one input to be read alongside clinical, biochemical, and radiological findings, not as a stand-alone diagnostic threshold [3].

The clinical value of this study lies in the description of a real-world cohort in which diagnostic classification, endocrine and radiological findings, GH1 sequence variation, and longitudinal outcomes during treatment were examined together. Rather than testing biological equivalence or establishing the predictive value of GH1 variants, this exploratory analysis describes whether routinely collected endocrine and genetic features were associated with observed growth outcomes and identifies limitations that should guide larger prospective studies.

Sequencing has broadened the genetic assessment of SS. Multigene panels and whole-exome sequencing have clarified much of its genetic basis, but we deliberately restricted the present analysis to the *GH1* gene, given its direct role in GH biology and in isolated GHD [5,6,7]. Pathogenic *GH1* variants are uncommon, and how *GH1* sequence variation relates to endocrine phenotype and to the long-term response to recombinant GH is still poorly defined [5,8,9]. Against this background, we characterized the clinical, endocrine, radiological, and *GH1* genetic features of children receiving GH therapy and asked whether *GH1* sequence variation was associated with five-year growth outcomes.

## 2. Materials and Methods

This retrospective cohort study included 74 children treated with GH for SS at a tertiary pediatric endocrinology clinic between 2017 and 2021. The institutional ethics committee approved the study (Approval no. 199; 2021, Date 30 June 2021), which followed the Declaration of Helsinki. Because the design was retrospective, the requirement for informed consent was waived.

**Figure 1:**
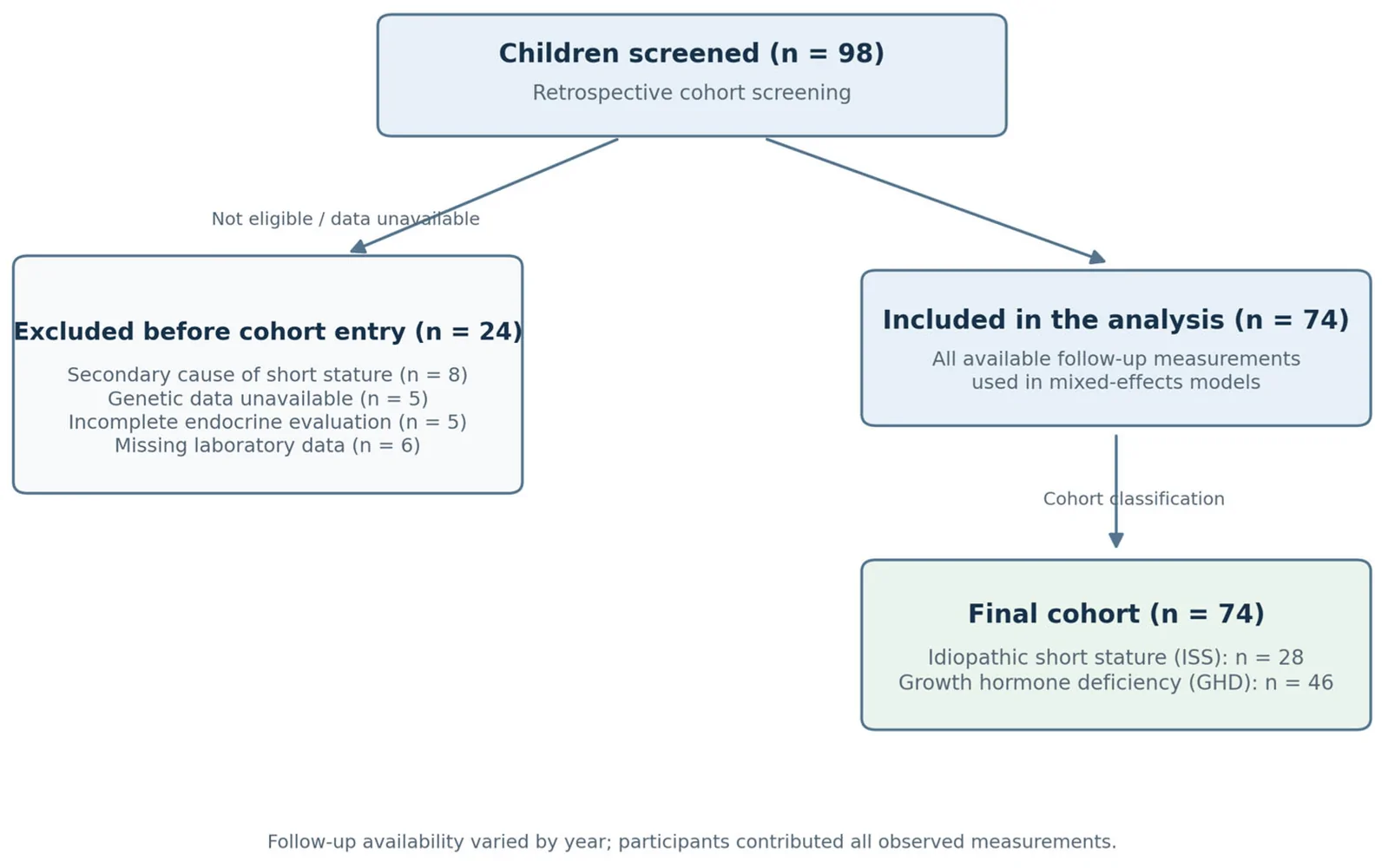
Flow chart of the study.

All 28 children classified as ISS had documented GH stimulation-test results, and all had a peak GH concentration ≥8 ng/mL. In the GHD group, archived stimulation-test results were available for 8 of 46 children, and all 8 had peak GH concentrations below the study cutoff of 8 ng/mL. The remaining 38 children were classified as GHD based on the integrated clinical, auxological, biochemical, radiological, and treatment-eligibility assessments documented in their medical records; GH stimulation-test results were unavailable for these patients. Because this was a retrospective study, the records did not permit reliable patient-level enumeration of how many of these 38 children fulfilled each individual component of the integrated assessment. This split was applied in line with recommendations that stimulation results be read together with auxological, biochemical, radiological, and clinical findings rather than in isolation [3,10]. Of 98 children screened, 24 were excluded before cohort entry: 8 because of a secondary cause of SS, 5 because genetic data were unavailable, 5 because the endocrine evaluation was incomplete, and 6 because laboratory data were missing. The remaining 74 children—28 classified as ISS and 46 as GHD—were included in the analysis (Figure 1). Follow-up measurements were incomplete for some participants, so the number contributing data varied by year and outcome. All available repeated measurements were included in the linear mixed-effects models; no imputation was performed. Complete measurements at all five annual assessment points were available for 16 participants.

Baseline demographic, clinical, endocrine, radiological, genetic, and treatment data were drawn from electronic medical records. Clinical variables were sex, pretreatment height, weight and height SDS, pituitary MRI findings, pituitary height, hypothyroidism during follow-up, and annual GH dose. Height, weight, and height SDS were derived from the Turkish Paediatric Endocrinology and Diabetes Association (ÇEDD) reference standards [11]. Abnormal pituitary MRI included pituitary hypoplasia, ectopic posterior pituitary, empty sella, or other structural changes.

Endocrine assessment covered baseline GH, peak GH responses to L-Dopa and clonidine, IGF-1, thyroid-stimulating hormone (TSH), free thyroxine (fT4), fasting glucose, follicle-stimulating hormone (FSH), luteinizing hormone (LH), estradiol, and testicular volume. For follow-up, we recorded the highest IGF-1, the lowest TSH and fT4, and the lowest and highest glucose values. Measurements were part of routine care, and stimulation results were interpreted together with growth velocity, pubertal status, IGF-1, and the overall clinical picture. Values considered implausible were checked against the original electronic medical records and corrected only when a documented transcription or data-entry error was confirmed; no observations were excluded solely because of their numerical value. Archived GH stimulation-test records were available for 36 patients overall: all 28 patients in the ISS group and 8 of 46 patients in the GHD group. The number of participants with available results varied across tests and measurements; the available sample size is reported for each variable. All available results were interpreted alongside the clinical, auxological, biochemical, and radiological findings.

GH therapy began after full endocrine evaluation, following routine practice. The starting dose was weight-adjusted to pretreatment body weight, whereas follow-up doses are given as absolute daily doses because serial weight-adjusted values were not consistently available. Treatment decisions took account of height below −2 SDS, reduced growth velocity, delayed bone age where present, IGF-1, pituitary imaging, and stimulation results. In ISS, recombinant GH was started only after identifiable causes had been excluded and after multidisciplinary endocrine review, in keeping with national reimbursement rules and international guidelines [3,10].

### 2.1. Genetic Analysis

*GH1* sequencing was performed on genomic DNA using the Illumina MiSeq platform. Reads were aligned to the GRCh37 (hg19) reference genome, and variants were called and annotated with a standard bioinformatics workflow. Each variant was categorized by its position relative to *GH1* (promoter/upstream, 5′ untranslated region [UTR], intronic, exonic, or downstream) and by zygosity and predicted functional consequence. Clinical significance was assigned using ACMG/AMP criteria [12] with reference to the ClinVar and VarSome databases. Because neither multigene panel testing nor whole-exome sequencing was performed, the genetic analysis was confined to *GH1* sequence variation.

The primary outcomes were longitudinal changes in height, weight, height SDS, annual height velocity, height gain, and weight gain over five years of GH therapy.

### 2.2. Statistical Analysis

Analyses were run in R 4.5.2. Continuous variables are summarized as mean ± standard deviation or median (interquartile range [IQR]), as appropriate. Categorical variables are summarized as frequencies and percentages. Groups were compared with Student’s *t*-test or the Wilcoxon rank-sum test for continuous variables and with Pearson’s chi-square or Fisher’s exact test for categorical variables. Longitudinal outcomes were analyzed using linear mixed-effects models, which included all available repeated measurements rather than restricting the analysis to participants with complete five-year follow-up. Follow-up year, treatment group, and the year-by-group interaction were included as fixed effects, with year 1 and the ISS group as reference categories. Models were adjusted for age at treatment initiation, sex, initial GH dose, and pretreatment height SDS. Participant-level random effects were used to account for within-person correlation. Model-based estimates and 95% confidence intervals are reported. The number of available observations at each time point is shown in Supplementary Table S1. Tukey-adjusted pairwise comparisons are presented as exploratory analyses in Supplementary Table S2. Because these comparisons were not restricted to the complete-case subset and the availability of annual measurements could vary over time, they were interpreted descriptively and were not used for the primary longitudinal inference. Genetic analyses used correspondence analysis, multiple correspondence analysis, k-means clustering, and hierarchical clustering based on Jaccard similarity, with the optimal cluster number set by the average silhouette width. Associations between genetic clusters and clinical variables were tested with Pearson’s chi-square or Fisher’s exact test. A sensitivity analysis restricted to children with archived GH stimulation-test results was considered; however, it was not performed because only eight children in the GHD group had retrievable stimulation-test records, precluding a meaningful and stable between-group comparison. No formal adjustment for multiple comparisons was applied to secondary and exploratory analyses; therefore, isolated nominally significant findings, including the Kowarski-test and sex-stratified results, should be interpreted as hypothesis-generating. A two-sided *p* < 0.05 was considered significant.

## 3. Results

The final analytic cohort comprised 74 children, including 22 girls (29.7%) and 52 boys (70.3%). The median age at treatment initiation was 11.43 years (IQR, 8.34–13.76). Twenty-eight children (37.8%) were classified as having ISS, and 46 (62.2%) as having GHD. Patient selection is summarized in Figure 1, and baseline demographic, clinical, and diagnostic characteristics are presented in Table 1.

A comparison of clinical, genetic, biochemical, and treatment characteristics by sex is presented in Supplementary Table S3.

Archived GH stimulation-test records were available for 36 patients overall: all 28 patients in the ISS group and 8 of 46 patients in the GHD group. The number with an available result varied by test and measurement; test-specific denominators are reported in Table 2. Because test availability differed between groups, a sensitivity analysis restricted to children with archived stimulation-test results was not performed (Table 1).

Treatment and endocrine characteristics are presented in Table 2. The median initial weight-adjusted GH dose was 0.03 mg/kg/day. Peak GH concentrations after L-Dopa and clonidine stimulation were lower in the GHD group than in the ISS group (all *p* < 0.001; Table 2). This difference is consistent with the diagnostic classification and should not be interpreted as an independent finding. Its interpretation is limited because archived stimulation-test results were available for only eight children in the GHD group; results were unavailable for the remaining 38.

A positive Kowarski test was recorded in 8 of 28 children with ISS (28.6%) and 1 of 46 children with GHD (2.2%; *p* = 0.001). As only nine children had a positive result, this finding should be interpreted as exploratory (Table 1).

The number of available observations decreased over follow-up. For height and weight, measurements were available for 73 children in Year 1, 61 in Year 2, 45 in Year 3, 27 in Year 4, and 16 in Year 5. The corresponding numbers for annual height gain were 72, 60, 43, 26, and 15; annual weight-gain measurements were available for 73, 60, 43, 26, and 15 children, respectively (Supplementary Table S1).

The linear mixed-effects models included 222 observations from 73 children for height and weight, 216 observations from 72 children for annual height gain, and 217 observations from 73 children for annual weight gain. Adjusted models included age at treatment initiation, sex, initial GH dose, and pretreatment height standard deviation score (SDS). Model estimates and overall tests are presented in Table 3 and Supplementary Table S4.

In adjusted models, height and weight increased over time (both overall time effects *p* < 0.001). Compared with Year 1, adjusted height was higher by 8.31 cm in Year 2, 15.88 cm in Year 3, 21.76 cm in Year 4, and 27.16 cm in Year 5 (all *p* < 0.001). Adjusted weight was higher than in Year 1 by 5.36 kg in Year 2, 10.82 kg in Year 3, 14.65 kg in Year 4, and 15.84 kg in Year 5 (all *p* < 0.001). The overall treatment-group effects and time-by-group interactions were not statistically significant for height or weight (Table 3; Supplementary Table S4).

**Figure 2:**
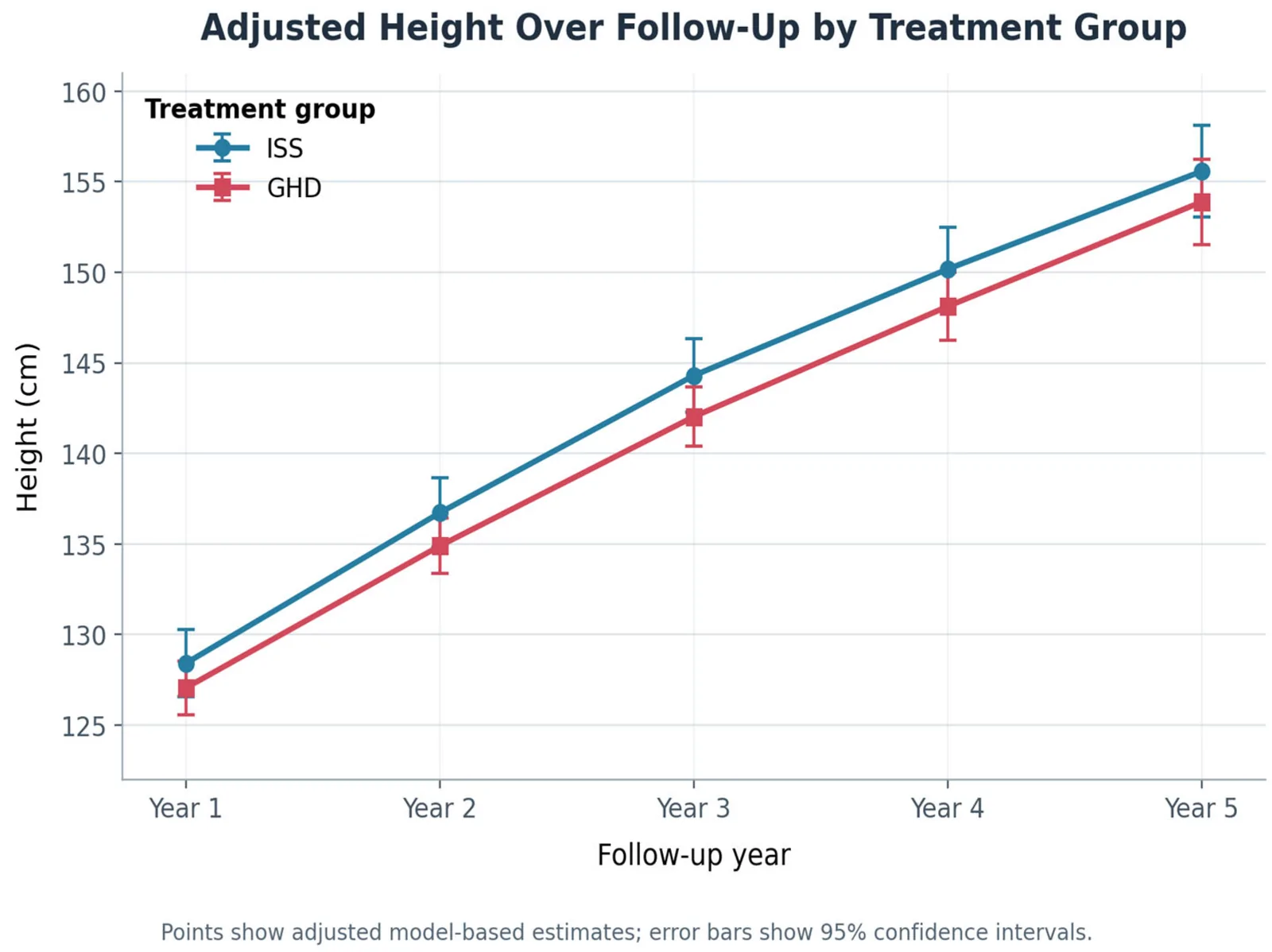
Adjusted model-based height by follow-up year and treatment group.

**Figure 3:**
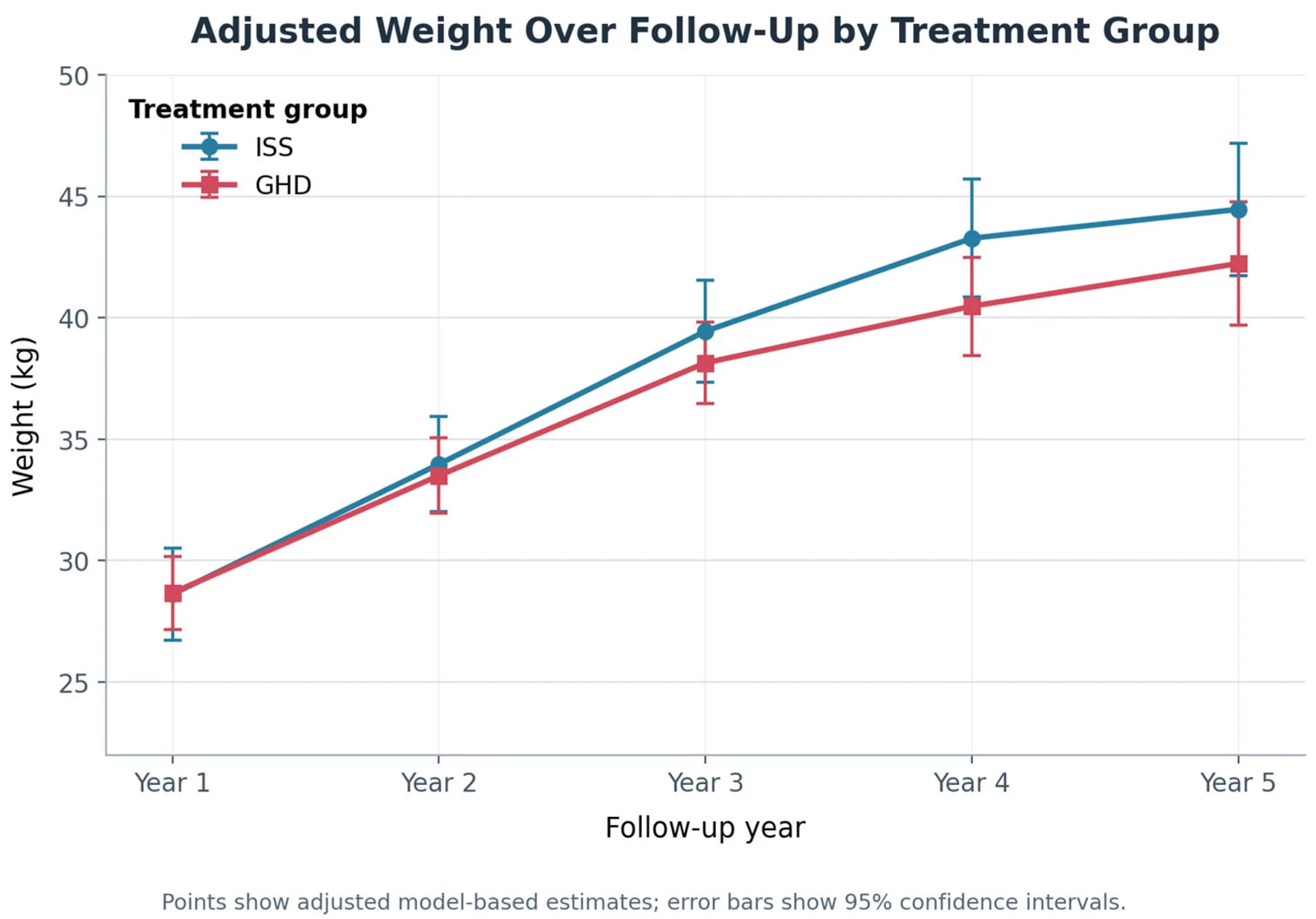
Adjusted model-based weight by follow-up year and treatment group. The x-axis shows follow-up year, and the y-axis shows weight (kg). Lines show adjusted estimates from the linear mixed-effects model; error bars indicate 95% CIs. Available observations at Years 1–5 were 73 (ISS 27, GHD 46), 61 (ISS 24, GHD 37), 45 (ISS 18, GHD 27), 27 (ISS 11, GHD 16), and 16 (ISS 8, GHD 8), respectively. The model was adjusted for age at treatment initiation, sex, initial GH dose, and pretreatment height SDS. The adjusted model showed an overall time effect (*p* < 0.001), but no significant treatment-group effect (*p* = 0.964) or time-by-group interaction (*p* = 0.340).

Annual height gain was lower in Year 4 and Year 5 than in Year 1, with adjusted differences of −3.38 cm/year and −4.62 cm/year, respectively (both *p* < 0.001). The differences between Year 1 and Year 2 or Year 3 were not statistically significant (*p* = 0.335 and *p* = 0.087, respectively). For annual weight gain, neither the overall time effect nor the individual contrasts between Year 1 and subsequent years were statistically significant. No statistically significant overall treatment-group effect or time-by-group interaction was detected for either annual growth outcome (Table 3; Supplementary Table S4). Adjusted model-based estimates by follow-up year and treatment group are provided in Supplementary Table S1. Adjusted height trajectories by treatment group are shown in Figure 2, and adjusted weight trajectories by treatment group are shown in Figure 3. Annual height-gain patterns by treatment group are shown in Supplementary Figure S1, while annual weight-gain patterns by sex are shown in Supplementary Figure S2. These figures are descriptive; no sex-by-time mixed-effects test was available for the sex-stratified trajectories.

The x-axis shows follow-up year, and the y-axis shows height (cm). Lines show adjusted estimates from the linear mixed-effects model; error bars indicate 95% confidence intervals (CIs). Available observations at Years 1–5 were 73 (ISS 27, GHD 46), 61 (ISS 24, GHD 37), 45 (ISS 18, GHD 27), 27 (ISS 11, GHD 16), and 16 (ISS 8, GHD 8), respectively. The model was adjusted for age at treatment initiation, sex, initial GH dose, and pretreatment height standard deviation score (SDS). The adjusted model showed an overall time effect (*p* < 0.001), but no significant treatment-group effect (*p* = 0.228) or time-by-group interaction (*p* = 0.941).

Baseline characteristics differed between participants with complete and incomplete five-year follow-up (Supplementary Table S5). Compared with participants with incomplete follow-up, those with complete follow-up were younger at treatment initiation (6.87 ± 2.55 vs. 12.58 years [IQR, 10.04–14.16]; *p* < 0.001) and had lower pretreatment height (101.14 ± 17.22 vs. 132.00 cm [IQR, 119.70–143.80]; *p* < 0.001), weight (16.02 ± 6.04 vs. 29.00 kg [IQR, 21.70–38.00]; *p* < 0.001), height SDS (−3.20 [IQR, −4.05 to −2.79] vs. −2.55 [IQR, −2.82 to −2.50]; *p* < 0.001), and baseline IGF-1 (89.19 ± 56.19 vs. 148.50 µg/L [IQR, 82.47–229.00]; *p* = 0.008). The initial weight-adjusted GH dose did not differ significantly between participants with complete and incomplete follow-up (*p* = 0.105). Sex distribution, treatment group, and pituitary MRI findings did not differ significantly. These differences indicate that participants with complete follow-up were not representative of the full cohort; estimates for later follow-up years should therefore be interpreted cautiously.

At least one GH1 sequence variant was identified in 70 of 74 children (94.6%); no variant was identified in 4 children (5.4%). Promoter/upstream variants were present in 67 children (90.5%), intronic variants in 54 (73.0%), 5′ untranslated region (UTR) variants in 21 (28.4%), exonic variants in 7 (9.5%), and downstream variants in 3 (4.1%). These categories were not mutually exclusive because some children carried variants in more than one region. No variant was classified as pathogenic or likely pathogenic. The main findings are summarized in Table 4, with variant-level details in Supplementary Table S6.

GH1 variant clustering was exploratory. The multiple correspondence analysis (MCA)-based k-means solution comprised clusters of 71, 2, and 1 participants. The average silhouette coefficients were 0.681 for MCA-based k-means clustering and 0.306 for Jaccard-based hierarchical clustering. Because the MCA-based clusters were highly imbalanced, they should not be interpreted as distinct biological subgroups. Clustering results are summarized in Table 5 and Supplementary Table S7; the MCA-based visualization is shown in Supplementary Figure S3.

## 4. Discussion

We followed children receiving recombinant GH for five years and integrated their clinical, endocrine, radiological, genetic, and growth data. This retrospective cohort provides real-world descriptive information on children classified as ISS or GHD in routine practice. Adjusted models did not detect statistically significant differences in height or weight trajectories between the groups, and GH1 sequence variation showed no clinically meaningful association with growth outcomes. These findings do not establish equivalent treatment efficacy or biological equivalence.

The similar growth of ISS and GHD children is the central observation. No statistically significant difference in the observed trajectories was detected. Moreover, the incomplete availability of archived stimulation-test records, particularly in the GHD group, prevented a meaningful test-restricted sensitivity analysis. The main comparison should therefore be interpreted in the context of the integrated clinical classification used in routine practice. In this treated cohort, the adjusted models did not detect a statistically significant difference in height or weight trajectories between children classified as ISS and GHD. Because the study was retrospective, lacked an untreated comparator, and was not designed to test equivalence, this result should be interpreted as an absence of a detected difference rather than evidence of similar treatment efficacy. The findings support further prospective evaluation of how clinical and endocrine characteristics relate to growth during GH treatment. Two caveats temper the interpretation. Without an untreated comparison group, the gains over time cannot be attributed to GH alone, because normal maturation drives growth in the same direction. And in the absence of a predefined equivalence margin and a priori power calculation, the lack of a significant group difference in a sample of this size is compatible with, but does not prove, equivalent responsiveness.

These results align with evidence that the response to GH depends on more than peak secretion, including growth-plate sensitivity, genetic background, age at treatment start, nutrition, and IGF-1 responsiveness [3]. Recent work frames linear growth as the product of endocrine and genetic mechanisms acting together, which blurs the traditional line between ISS and GHD [3,5,13,14].

Height rose steadily across the five years, while weight gain flattened in the later years. The same shape appears in earlier longitudinal work: height velocity accelerates most in the first one to two years of therapy and then settles toward physiological rates, even as cumulative height continues to accrue [1,3,15].

Height gain SDS, annual height velocity, and mean post-treatment height and weight gains did not differ significantly between the ISS and GHD groups. Adjusted longitudinal models likewise showed no significant treatment-group effect or time-by-group interaction for height, weight, annual height gain, or annual weight gain. These findings should be interpreted cautiously because follow-up declined over time and the study was not designed to establish equivalent treatment efficacy. Although age at treatment initiation did not differ significantly between groups, unmeasured factors such as pubertal stage and progression, target height, bone age, and treatment adherence may have influenced individual growth outcomes.

Sex differences warrant a brief note. Boys gained more in annual height and received higher absolute GH doses during follow-up. Since GH is dosed by body weight and boys were generally heavier, the higher absolute doses were expected and do not represent greater pharmacological exposure. The differences most likely reflect body size and growth potential rather than a true sex effect on responsiveness, though confirmation would require adjustment for longitudinal weight.

The safety signal was favorable. Baseline and follow-up IGF-1, thyroid function, glucose, gonadotropins, and most biochemical measures were comparable in ISS and GHD, and hypothyroidism arose in only a few children. This is consistent with the established safety of recombinant GH under routine endocrine monitoring [3,10].

One finding invites further study: positive Kowarski tests clustered in the ISS group. Because the Kowarski test played no part in the diagnostic classification, this pattern is not an artifact of group definition. It raises the possibility that some children labeled ISS have subtle neurosecretory dysfunction despite normal stimulated GH [3,16]. With only nine positive results, the observation is hypothesis-generating and needs confirmation in larger prospective cohorts.

The *GH1* analysis was, in effect, negative. Most variants were common promoter/upstream and intronic polymorphisms; coding variants were rare, and none reached pathogenic or likely pathogenic status under ACMG/AMP [12]. No meaningful association emerged between *GH1* variant patterns and endocrine phenotype, treatment group, sex, or growth. Given this composition, the clustering is exploratory rather than evidence of distinct genetic subtypes. The result does not argue against a genetic contribution to SS; it fits the understanding that linear growth is governed by many genes together with growth-plate biology, endocrine pathways, extracellular-matrix organization, and environment [1,5]. Variation within *GH1* alone is therefore unlikely to account for the clinical heterogeneity or the long-term response seen here.

This is in keeping with genomic studies in which the diagnostic yield of genetic testing is highest in carefully phenotyped children with syndromic features or familial SS [9,17,18]. Pathogenic variants in *NPR2*, *ACAN*, *SHOX*, and *HMGA2* have been reported in selected patients [5,17,19,20,21], but those genes were outside our scope. Our data should thus be read not as excluding a genetic basis for SS, but as showing that *GH1* sequence variation alone did not explain the heterogeneity in this cohort.

The study has several strengths. Longitudinal auxological data, endocrine assessment, pituitary imaging, biochemical monitoring, and genetic analysis were integrated in a single cohort. Five years of follow-up allowed us to describe growth beyond the first year of treatment, and linear mixed-effects models incorporated all available repeated measurements to characterize growth trajectories and explore potential genotype–phenotype associations.

Several limitations should be considered when interpreting these findings. First, the retrospective, single-center design and modest sample size limited statistical power, particularly for subgroup and genetic analyses. Second, follow-up was incomplete: only 16 participants had measurements at all five annual assessment points, participants with complete and incomplete follow-up differed in several baseline characteristics, and the number of observations declined over time. Although the linear mixed-effects models included all available measurements, the follow-up pattern may have been informative; estimates for later years should therefore be interpreted cautiously. Third, archived GH stimulation-test results were available for all children classified as ISS but for only eight children classified as GHD. This imbalance prevented a meaningful sensitivity analysis restricted to patients with documented test results. The remaining 38 children in the GHD group were classified using integrated clinical, auxological, biochemical, radiological, and treatment-eligibility assessments documented in their medical records. Because the study was retrospective, the records did not allow us to reliably determine how many of these children met each individual component of the assessment. Fourth, genetic testing was limited to *GH1*; variants in growth-plate and skeletal genes such as *NPR2*, *ACAN*, and *SHOX* were not assessed, and the detected variants were not functionally validated. Finally, target height, bone age at treatment initiation, pubertal stage and progression, nutritional status, and treatment adherence were not fully captured. These factors may have influenced individual growth outcomes and contributed to the observed variation in height and weight trajectories.

Multicenter studies with broader genomic testing and functional validation are needed to map the mechanisms behind pediatric SS and to refine individualized GH treatment. Bibliometric data point the field in the same direction, toward precision medicine and multi-omics [22]. In time, models that combine clinical, endocrine, and molecular information may support more personalized GH strategies and better long-term growth [5,13].

## 5. Conclusions

In this retrospective exploratory cohort, adjusted models did not detect significant differences in height or weight trajectories between GH-treated children classified as ISS or GHD. This finding does not establish equivalent treatment efficacy. GH1 sequence variation was not meaningfully associated with the observed endocrine phenotype or growth outcomes. Larger prospective studies with broader genetic testing are needed.

## Figures and Tables

**Table 1:** Baseline Demographic, Clinical, and Diagnostic Characteristics by Treatment Group.

Variable	Available n (ISS/GHD)	ISS (n = 28)	GHD (n = 46)	*p* Value
**Age at treatment initiation, years**	28/46	10.77 (4.95–15.88)	11.64 (1.79–15.31)	0.321
**Female sex**	28/46	10/28 (35.7)	12/46 (26.1)	0.538
**Male sex**	28/46	18/28 (64.3)	34/46 (73.9)	
**Pretreatment height, cm**	28/45	126.6 (77.0–148.8)	126.0 (68.0–150.5)	0.548
**Pretreatment height SDS**	28/44	−2.65 (−7.50 to −1.89)	−2.64 (−6.69 to −1.74)	0.835
**Pretreatment weight, kg**	28/45	24.9 (8.4–39.4)	29.0 (6.8–74.0)	0.196
**Pituitary MRI, normal**	28/45	26/28 (92.9)	37/45 (82.2)	0.299
**Pituitary MRI, abnormal**	28/45	2/28 (7.1)	8/45 (17.8)	
**Pituitary height, mm**	15/21	4.8 (3.0–7.0)	4.0 (1.33–9.5)	0.101
**Kowarski test positive**	28/46	8/28 (28.6)	1/46 (2.2)	**0.001**
**GH stimulation test performed**	28/46	28/28 (100)	8/46 (17.4)	**<0.001**
**ISS group**		28 (100)	—	—
**GHD group**		—	46 (100)	—

Note. Continuous variables are reported as mean ± SD for approximately normally distributed data and as median (IQR) for skewed data. The “Available n” column indicates the number of participants with data available for each variable. MRI findings were available for 73 participants; the GHD denominator for MRI findings was 45. GH stimulation-test results were available for all 28 participants in the ISS group and 8 of 46 participants in the GHD group. Treatment-group rows describe the cohort classification and are not separate comparisons. Reference intervals for pituitary height and endocrine measurements are not provided because the retrospective records did not consistently document the imaging reference standard, assay platform, or age- and pubertal-stage-specific laboratory intervals used at the time of testing. These values are therefore presented descriptively. Bold formatting in the *p* value column indicates statistical significance (*p* < 0.05). Variable names are bolded for readability. “–” indicates that the value is not applicable; no between-group comparison was performed for the ISS and GHD classification rows. Thank you for pointing this out. We have clarified in the table footnote that “–” indicates that the value is not applicable and that no between-group comparison was performed for the ISS and GHD classification rows. Abbreviations: GHD, growth hormone deficiency; GH, growth hormone; ISS, idiopathic short stature; MRI, magnetic resonance imaging; SDS, standard deviation score.

**Table 2:** Growth Hormone Treatment, Endocrine Parameters, and Growth Outcomes by Treatment Group.

Parameter	Available n (ISS/GHD)	ISS (n = 28)	GHD (n = 46)	*p* Value
**Initial GH dose, mg/kg/day**	28/45	0.03 (0.01–0.04)	0.03 (0.00–0.10)	0.165
**Year 2 GH dose, mg/day**	26/39	2.95 (1.06–4.30)	3.20 (1.29–6.80)	0.108
**Year 3 GH dose, mg/day**	23/31	3.60 (1.50–4.80)	3.20 (1.50–7.40)	0.500
**Year 4 GH dose, mg/day**	16/21	3.65 (1.60–6.00)	3.20 (1.50–8.80)	0.712
**Year 5 GH dose, mg/day**	10/11	3.50 (2.00–5.70)	3.00 (2.00–5.20)	0.416
**Annual mean GH dose, mg/day**	28/46	3.15 (1.56–4.40)	3.68 (1.33–6.90)	0.089
**Baseline GH, ng/mL**	18/19	0.50 (0.05–25.0)	0.37 (0.06–4.51)	0.659
**L-Dopa GH peak 1, ng/mL**	27/8	5.02 (0.05–26.25)	0.77 (0–7.49)	**<0.001**
**L-Dopa GH peak 2, ng/mL**	27/8	4.48 (1.24–26.07)	1.42 (0.01–7.75)	**<0.001**
**Clonidine GH peak 1, ng/mL**	20/8	9.20 (0.05–21.3)	4.18 (0.02–7.68)	**<0.001**
**Clonidine GH peak 2, ng/mL**	20/8	6.88 (0.05–19.2)	3.22 (0.02–7.05)	**<0.001**
**Baseline IGF-1, µg/L**	28/44	144.25 (25–432)	99.15 (15–487)	0.206
**Final IGF-1, µg/L**	28/46	322.5 (50.4–676)	298 (31.8–569)	0.493
**Highest IGF-1, µg/L**	28/46	379 (136–676)	329 (59.1–674)	0.545
**Baseline TSH, µU/mL**	24/41	2.02 (1.2–5.54)	2.30 (0–9.2)	0.178
**Baseline free thyroxine, ng/dL**	25/43	1.00 (0.39–1.34)	0.92 (0.30–1.32)	0.225
**Baseline glucose, mg/dL**	28/42	85.5 (69–107)	87.5 (73–131)	0.569
**Baseline FSH, mIU/mL**	3/5	1.20 (0.4–5.0)	1.50 (1.4–2.1)	0.551
**Baseline LH, mIU/mL**	8/8	0.24 (0.09–0.44)	0.60 (0.01–1.61)	0.066
**Annual height velocity, cm/year**	27/45	8.7 (3.2–13.5)	9.2 (2.7–19.2)	0.460
**Height gain SDS**	27/45	1.41 (−0.89 to 6.52)	1.88 (−2.21 to 8.18)	0.866
**Mean post-treatment height gain, cm/year**	27/45	7.5 (3.3–11.4)	7.5 (4.8–14.5)	0.963
**Mean post-treatment weight gain, kg/year**	27/46	4.8 (1.8–9.5)	5.6 (0–15.9)	0.932

Note. Continuous variables are reported as mean ± SD for approximately normally distributed data and as median (IQR) for skewed data. “Available n (ISS/GHD)” gives the number of participants with a non-missing value in each group. Initial GH dose is expressed per kilogram of body weight per day; subsequent and annual mean GH doses are expressed as absolute daily doses. GH stimulation-test results were available for all 28 participants in the ISS group and for 8 of 46 participants in the GHD group; the reported test results and corresponding *p* values are based on participants with available results. Other variable-specific denominators may vary because of missing data. Abbreviations: FSH, follicle-stimulating hormone; GH, growth hormone; GHD, growth hormone deficiency; IGF-1, insulin-like growth factor 1; ISS, idiopathic short stature; LH, luteinizing hormone; SDS, standard deviation score; TSH, thyroid-stimulating hormone.

**Table 3:** Adjusted Linear Mixed-Effects Model Results for Longitudinal Growth Outcomes and Annual Growth Gain.

Outcome	Overall Time *p*	Overall Group *p*	Time × Group *p*	Selected Adjusted Contrasts vs. Year 1
**Height**	**<0.001**	0.228	0.941	Year 5 vs. Year 1: +27.16 cm (24.93 to 29.39), *p* < 0.001
**Weight**	**<0.001**	0.964	0.340	Year 5 vs. Year 1: +15.84 kg (13.31 to 18.37), *p* < 0.001
**Annual height gain**	**<0.001**	0.653	0.853	Year 4: −3.38 cm (−5.19 to −1.56); Year 5: −4.62 cm (−6.67 to −2.57), both *p* < 0.001
**Annual weight gain**	0.169	0.510	0.392	No year contrast vs. Year 1 was significant

Note: Overall *p* values for time, treatment group, and the time-by-group interaction are from adjusted linear mixed-effects models. Models were adjusted for age at treatment initiation, sex, initial GH dose, and pretreatment height SDS; Year 1 and the ISS group were the reference categories. Selected contrasts are model-based adjusted differences from Year 1 and are reported with 95% confidence intervals. Annual height gain is expressed in cm/year and annual weight gain in kg/year. No statistically significant time-by-group interaction was detected; this finding does not establish equivalence between groups. Abbreviations: GH, growth hormone; GHD, growth hormone deficiency; ISS, idiopathic short stature; SDS, standard deviation score.

**Table 4:** Summary of GH1 Variant and Clustering Findings.

Finding	Result	Interpretation
**At least one GH1 variant**	70/74 (94.6%)	No variant: 4/74 (5.4%)
**Promoter/upstream region**	67/74 (90.5%)	Overlapping categories; not mutually exclusive
**Intronic region**	54/74 (73.0%)	Overlapping categories; not mutually exclusive
**5′ UTR region**	21/74 (28.4%)	Overlapping categories; not mutually exclusive
**Exonic variant**	7/74 (9.5%)	Coding variants uncommon
**Downstream region**	3/74 (4.1%)	Overlapping categories; not mutually exclusive
**Pathogenic/likely pathogenic variant**	0	No such variants identified
**Clustering**	Exploratory	No clinically meaningful pattern identified

Note. Values are presented as n (%). Percentages are based on the full cohort (N = 74). Participants could carry multiple GH1 variants; therefore, genomic-region categories are not mutually exclusive and their percentages do not sum to 100%. No pathogenic or likely pathogenic variants were identified according to ACMG/AMP classification. Clustering analyses were exploratory and did not identify a clinically meaningful pattern. Abbreviations: GH1, growth hormone 1; UTR, untranslated region.

**Table 5:** Summary of genetic clustering analyses.

Parameter	Result
Optimal cluster number	3
MCA Dim1 contribution	9.9%
MCA Dim2 contribution	8.7%
Average silhouette coefficient (for Jaccard + hierarchical clustering)	0.306
Average silhouette coefficient (for MCA + k-means clustering)	0.681

Note. Three clusters were selected for both the Jaccard similarity-based hierarchical clustering and the MCA-based k-means clustering. The average silhouette coefficients correspond to these methods, respectively. MCA dimension contributions indicate the proportion of inertia explained by each dimension. Clustering analyses were exploratory and should not be interpreted as evidence of distinct biological subgroups. Abbreviations: MCA, multiple correspondence analysis.

## Data Availability

The data that support the findings of this study are available from the corresponding author upon reasonable request. The data are not publicly available because they contain information derived from individual medical records and are subject to privacy and ethical restrictions.

## Supplementary Materials

The following supporting information can be downloaded at: https://www.mdpi.com/article/10.3390/genes17101279/s1, Supplementary Table S1: Available Ob-servations and Adjusted Model-Based Estimates by Follow-Up Year; Supplementary Table S2: Ex-ploratory Tukey-Adjusted Pairwise Comparisons of Annual Height and Weight; Supplementary Table S3. Comparison of Clinical, Genetic, Biochemical, and Treatment Characteristics by Sex; Supplementary Table S4: Adjusted Fixed-Effect Estimates and Overall Tests from Linear Mixed-Effects Models; Supplementary Table S5: Baseline Characteristics by Follow-Up Complete-ness; Supplementary Table S6: Frequency, Genomic Location, Zygosity, and ACMG/AMP Classi-fication of GH1 Sequence Variants; Supplementary Table S7: Descriptive Summary of Exploratory GH1 Variant Clustering Analyses; Supplementary Figure S1: Five-Year Annual Height Gain by Treatment Group; Supplementary Figure S2: Five-Year Annual Weight Gain by Gender; and Sup-plementary Figure S3: MCA-Based Visualization of GH1 Variant Clusters.

## Abbreviations

The following abbreviations are used in this manuscript:

ACAN	Aggrecan gene
ACMG/AMP	American College of Medical Genetics and Genomics/Association for Molecular Pathology
CA	Correspondence analysis
DNA	Deoxyribonucleic acid
dbSNP	Database of Single Nucleotide Polymorphisms
fT4	Free thyroxine
FSH	Follicle-stimulating hormone
GH	Growth hormone
GH1	Growth hormone 1 gene
GHD	Growth hormone deficiency
GHI	Growth hormone insensitivity
GRCh37	Genome Reference Consortium human genome build 37
HGVS	Human Genome Variation Society
HMGA2	High-mobility group AT-hook 2 gene
IGF-1	Insulin-like growth factor 1
ISS	Idiopathic short stature
L-Dopa	L-3,4-dihydroxyphenylalanine (levodopa)
LH	Luteinizing hormone
MCA	Multiple correspondence analysis
MRI	Magnetic resonance imaging
NPR2	Natriuretic peptide receptor 2 gene
SDS	Standard deviation score
SE	Standard error
SHOX	Short stature homeobox gene
SNV	Single-nucleotide variant
SS	Short stature
TSH	Thyroid-stimulating hormone
UTR	Untranslated region
hg19	Human genome build 19
NS	Not significant
Het	Heterozygous
Hom	Homozygous
