# Supplementary material for: Endocrine Phenotype and GH1 Sequence Variation in Children with Short Stature: Five-Year Growth Outcomes of Growth Hormone Therapy
Source: Genes (Basel). 2026 Oct 14;17(10):1279. doi: 10.3390/genes17101279 (PMC13703992; doi:10.3390/genes17101279)
Supplement: Supplementary file 1 [file genes-17-01279-s001.zip › genes-4584043-supplementary.pdf]

Supplementary Table S1. Available Observations and Adjusted Model-Based Estimates by Follow-Up Year

Outcome	Time	Observed n (ISS; GHD)	ISS estimate (95% CI)	GHD estimate (95% CI)
Height	Year 1	73 (27 ISS; 46 GHD)	128.42 (126.56 to 130.29)	127.06 (125.57 to 128.54)
Height	Year 2	61 (24 ISS; 37 GHD)	136.73 (134.82 to 138.64)	134.90 (133.38 to 136.41)
Height	Year 3	45 (18 ISS; 27 GHD)	144.30 (142.26 to 146.35)	142.03 (140.41 to 143.66)
Height	Year 4	27 (11 ISS; 16 GHD)	150.18 (147.88 to 152.49)	148.12 (146.23 to 150.02)
Height	Year 5	16 (8 ISS; 8 GHD)	155.58 (153.04 to 158.12)	153.89 (151.55 to 156.23)
Weight	Year 1	73 (27 ISS; 46 GHD)	28.62 (26.73 to 30.51)	28.67 (27.16 to 30.18)
Weight	Year 2	61 (24 ISS; 37 GHD)	33.97 (32.02 to 35.92)	33.50 (31.95 to 35.04)
Weight	Year 3	45 (18 ISS; 27 GHD)	39.44 (37.33 to 41.55)	38.14 (36.46 to 39.83)
Weight	Year 4	27 (11 ISS; 16 GHD)	43.27 (40.84 to 45.71)	40.47 (38.45 to 42.48)
Weight	Year 5	16 (8 ISS; 8 GHD)	44.46 (41.74 to 47.18)	42.23 (39.69 to 44.78)
Annual height gain	Year 1	72 (27 ISS; 45 GHD)	9.36 (8.34 to 10.37)	9.65 (8.82 to 10.48)
Annual height gain	Year 2	60 (24 ISS; 36 GHD)	8.66 (7.59 to 9.73)	8.41 (7.54 to 9.28)
Annual height gain	Year 3	43 (18 ISS; 25 GHD)	8.02 (6.79 to 9.25)	7.17 (6.13 to 8.21)
Annual height gain	Year 4	26 (11 ISS; 15 GHD)	5.98 (4.44 to 7.53)	6.09 (4.72 to 7.45)
Annual height gain	Year 5	15 (8 ISS; 7 GHD)	4.74 (2.90 to 6.57)	4.75 (2.82 to 6.69)
Annual weight gain	Year 1	73 (27 ISS; 46 GHD)	4.71 (3.55 to 5.87)	5.20 (4.27 to 6.12)
Annual weight gain	Year 2	60 (24 ISS; 36 GHD)	5.63 (4.41 to 6.86)	5.13 (4.13 to 6.12)
Annual weight gain	Year 3	43 (18 ISS; 25 GHD)	5.98 (4.57 to 7.38)	5.30 (4.11 to 6.49)
Annual weight gain	Year 4	26 (11 ISS; 15 GHD)	5.69 (3.92 to 7.45)	3.98 (2.42 to 5.54)
Annual weight gain	Year 5	15 (8 ISS; 7 GHD)	3.23 (1.14 to 5.33)	4.66 (2.45 to 6.87)

Note. Observed n indicates the number of participants with measurements available at each follow-up year; group-specific counts are shown in parentheses. Estimates are adjusted model-based means with 95% confidence intervals, not raw means. Models were adjusted for age at treatment initiation, sex, initial GH dose, and pretreatment height SDS. Height is reported in cm, weight in kg, annual height gain in cm/year, and annual weight gain in kg/year.

Abbreviations: CI, confidence interval; GHD, growth hormone deficiency; GH, growth hormone; ISS, idiopathic short stature; SDS, standard deviation score.

Supplementary Table S2. Exploratory Tukey-Adjusted Pairwise Comparisons of Annual Height and Weight

Outcome	Group	Comparison	Mean difference	SE	Adjusted P
Height	ISS	Year 1 vs Year 2	-8.242	0.783	**<0.001**
Height	ISS	Year 1 vs Year 3	-15.832	0.876	**<0.001**
Height	ISS	Year 1 vs Year 4	-21.788	1.055	**<0.001**
Height	ISS	Year 1 vs Year 5	-27.175	1.195	**<0.001**
Height	ISS	Year 2 vs Year 3	-7.590	0.876	**<0.001**
Height	ISS	Year 2 vs Year 4	-13.547	1.055	**<0.001**
Height	ISS	Year 2 vs Year 5	-18.933	1.195	**<0.001**
Height	ISS	Year 3 vs Year 4	-5.956	1.079	**<0.001**
Height	ISS	Year 3 vs Year 5	-11.343	1.216	**<0.001**
Height	ISS	Year 4 vs Year 5	-5.387	1.285	**<0.001**
Height	GHD	Year 1 vs Year 2	-7.914	0.631	**<0.001**
Height	GHD	Year 1 vs Year 3	-15.218	0.713	**<0.001**
Height	GHD	Year 1 vs Year 4	-21.385	0.871	**<0.001**
Height	GHD	Year 1 vs Year 5	-27.201	1.155	**<0.001**
Height	GHD	Year 2 vs Year 3	-7.304	0.713	**<0.001**
Height	GHD	Year 2 vs Year 4	-13.472	0.871	**<0.001**
Height	GHD	Year 2 vs Year 5	-19.288	1.155	**<0.001**
Height	GHD	Year 3 vs Year 4	-6.168	0.892	**<0.001**
Height	GHD	Year 3 vs Year 5	-11.983	1.170	**<0.001**
Height	GHD	Year 4 vs Year 5	-5.816	1.223	**<0.001**
Weight	ISS	Year 1 vs Year 2	-5.246	0.895	**<0.001**
Weight	ISS	Year 1 vs Year 3	-10.523	1.001	**<0.001**
Weight	ISS	Year 1 vs Year 4	-14.403	1.206	**<0.001**
Weight	ISS	Year 1 vs Year 5	-15.632	1.365	**<0.001**
Weight	ISS	Year 2 vs Year 3	-5.277	1.001	**<0.001**
Weight	ISS	Year 2 vs Year 4	-9.157	1.206	**<0.001**
Weight	ISS	Year 2 vs Year 5	-10.386	1.365	**<0.001**
Weight	ISS	Year 3 vs Year 4	-3.880	1.233	**0.017**
Weight	ISS	Year 3 vs Year 5	-5.109	1.390	**0.003**
Weight	ISS	Year 4 vs Year 5	-1.229	1.469	0.919
Weight	GHD	Year 1 vs Year 2	-5.054	0.721	**<0.001**
Weight	GHD	Year 1 vs Year 3	-9.317	0.815	**<0.001**
Weight	GHD	Year 1 vs Year 4	-12.627	0.995	**<0.001**
Weight	GHD	Year 1 vs Year 5	-14.307	1.320	**<0.001**
Weight	GHD	Year 2 vs Year 3	-4.263	0.815	**<0.001**
Weight	GHD	Year 2 vs Year 4	-7.573	0.995	**<0.001**
Weight	GHD	Year 2 vs Year 5	-9.253	1.320	**<0.001**
Weight	GHD	Year 3 vs Year 4	-3.309	1.019	**0.012**
Weight	GHD	Year 3 vs Year 5	-4.990	1.338	**0.003**
Weight	GHD	Year 4 vs Year 5	-1.680	1.397	0.750

Note. *Pairwise comparisons are Tukey-adjusted contrasts between follow-up years within each treatment group. Mean differences are calculated as the first time point minus the second time point in the comparison column; therefore, negative values indicate a higher estimated value at the later time point. SE denotes the standard error of the mean difference. These comparisons are exploratory and descriptive; the primary longitudinal inference is based on the adjusted linear mixed-effects models. Height is reported in cm and weight in kg.*

Abbreviations: *GHD, growth hormone deficiency; ISS, idiopathic short stature; SE, standard error*.

Supplementary Table S3. Comparison of Clinical, Genetic, Biochemical, and Treatment Characteristics by Sex

Characteristic	Female (n=22)	Male (n=52)	P value
**Treatment Group**			
ISS	10 (45.45)	18 (34.62)	0.538
GHD	12 (54.55)	34 (65.38)	
**Presence of downstream variant**	1 (4.55)	2 (3.85)	>0.999
**Intronic variant**	18 (81.82)	36 (69.23)	0.392
**Exonic variant**	2 (9.09)	5 (9.62)	>0.999
**5′ UTR variant**	8 (36.36)	13 (25.00)	0.478
**Heterozygous variant**	21 (95.45)	48 (92.31)	>0.999
**Homozygous variant**	9 (40.91)	25 (48.08)	0.756
**GH stimulation test performed**	12 (54.55)	24 (46.15)	0.685
**Pituitary MRI findings**			
Normal	18 (85.71)	45 (86.54)	>0.999
Abnormal	3 (14.29)	7 (13.46)	
**Pituitary height (mm)***	6.65 (3.0- 9.5)	4.05 (1.33 – 7.0)	**0.002**
**Kowarski test**	3 (13.64)	6 (11.54)	>0.999
**Complication (Hypothyroidism)**	5 (22.73)	2 (3.85)	**0.022**
**Pre-treatment height (cm)***	116 (86.2-150.5)	130.1 (68-150.5)	0.105
**Pre-treatment height SDS***	-3 (-4.5--2.31)	-2.55 (-7.5--1.74)	**0.011**
**Pre-treatment weight (kg)***	21 (12-60)	29 (6.8-74)	0.073
**Height gain SDS***	1.84 (-1.45-6.52)	1.78 (-2.21-8.18)	0.577
**Initial GH dose (mg/kg/day)***	0.03 (0.03-0.04)	0.03 (0.01-0.1)	0.275
**Second-year GH dose (mg/day)***	2.25 (1.3-4.4)	3.3 (1.06-6.8)	**0.004**
**Third-year GH dose (mg/day)***	2.4 (1.5-5)	3.9 (1.5-7.4)	**0.013**
**Fourth-year GH dose (mg/day)***	2.3 (1.6-3.9)	4.9 (1.5-8.8)	**0.006**
**Fifth-year GH dose (mg/day)***	2.9 (2-4.2)	3.3 (2-5.7)	0.287
**Annual mean GH dose (mg/day)***	2.42 (1.38-5.6)	3.67 (1.33-6.9)	**0.020**
**Baseline GH (ng/mL)***	0.21 (0.05-4.4)	0.55 (0.06-25)	**0.007**
**L-Dopa GH peak 1 (ng/mL)***	4.13 (0.09-14.02)	1.49 (0-26.25)	0.087
**L-Dopa GH peak 2 (ng/mL)***	4.55 (0.78-10.23)	1.93 (0.01-26.07)	**0.008**
**Clonidine GH peak 1 (ng/mL)***	5.16 (0.67-19.7)	5.4 (0.02-21.3)	0.706
**Clonidine GH peak 2 (ng/mL)***	4.42 (2.19-19.2)	4.39 (0.02-18.64)	0.475
**Baseline IGF-1 (μg/L)***	110.5 (32.6-487)	140.5 (15-432)	0.741
**Final IGF-1 (μg/L)***	298.64 ± 130.41	332.4 ± 149.35	0.360
**Highest IGF-1 (μg/L)***	325.43 ± 121.16	377.53 ± 154.05	0.163
**Baseline TSH (μU/mL)***	2.03 (0.98-9.24)	2.21 (0-5.54)	0.407
**First-year TSH (μU/mL)***	1.92 (0.03-6.89)	2.04 (0-6.91)	0.332

Characteristic	Female (n=22)	Male (n=52)	P value
**Baseline free thyroxine (ng/dL)***	0.88 ± 0.23	0.95 ± 0.21	0.226
**First-year free thyroxine (ng/dL)***	0.96 (0.32-1.22)	0.94 (0.63-2.09)	0.740
**Lowest free thyroxine (ng/dL)***	0.78 (0.3-1.22)	0.85 (0.51-1.32)	0.489
**Lowest TSH (μU/mL)***	1.04 (0.01-12.68)	1.62 (0-3.38)	0.064
**Baseline glucose (mg/dL)***	85.5 (69-107)	87 (71-131)	0.160
**Lowest glucose (mg/dL)***	78 (56-96)	82 (61-111)	0.052
**Highest glucose (mg/dL)***	96.5 (75-129)	97.5 (75-131)	0.884
**Baseline FSH (mIU/mL)***	1.39 (1.19-4.97)	1.48 (0.41-2.1)	>0.999
**First-year FSH (mIU/mL)***	0.38 (0.38-0.38)	1.86 (0.45-2.86)	0.295
**Baseline LH (mIU/mL)***	0.3 (0.17-0.59)	0.37 (0.01-1.61)	0.704
**First-year LH (mIU/mL)***	0.27 (0.2-0.36)	0.88 (0-2.06)	0.494
**Annual height velocity (cm/year)***	8.44 (3.18-12.32)	9.47 (2.71-19.2)	0.151
**Mean post-treatment height gain (cm/year)***	6.93 (3.3-14.5)	8 (5.7-13)	**0.004**
**Mean post-treatment weight gain (kg/year)***	3.85 (0-7.3)	5.7 (1.62-15.9)	**0.017**

Note. *Categorical variables are presented as n (%). Continuous variables are reported as mean ± SD for approximately normally distributed data and as median (IQR) for skewed data. Denominators vary where data were missing. Pituitary MRI findings were available for 21 women and 52 men. GH stimulation-test results were available for 36 participants overall. Participants could carry multiple GH1 variants; therefore, heterozygous and homozygous findings may overlap at the participant level.*

Abbreviations: *FSH, follicle-stimulating hormone; GH, growth hormone; GHD, growth hormone deficiency; IGF-1, insulin-like growth factor 1; ISS, idiopathic short stature; LH, luteinizing hormone; MRI, magnetic resonance imaging; SDS, standard deviation score; TSH, thyroid-stimulating hormone; UTR, untranslated region.*

Supplementary Table S4. Adjusted Fixed-Effect Estimates and Overall Tests from Linear Mixed-Effects Models

Outcome	Model term	Estimate (95% CI) or test	P value
Height	Year 2 vs Year 1	8.309 (6.842 to 9.777)	**<0.001**
Height	Year 3 vs Year 1	15.883 (14.251 to 17.516)	**<0.001**
Height	Year 4 vs Year 1	21.761 (19.795 to 23.728)	**<0.001**
Height	Year 5 vs Year 1	27.156 (24.927 to 29.386)	**<0.001**
Height	GHD vs ISS	−1.363 (−3.576 to 0.851)	0.228
Height	Year 2 × GHD	−0.470 (−2.359 to 1.420)	0.626
Height	Year 3 × GHD	−0.907 (−3.024 to 1.209)	0.401
Height	Year 4 × GHD	−0.696 (−3.270 to 1.878)	0.596
Height	Year 5 × GHD	−0.325 (−3.422 to 2.773)	0.837
Height	Age at treatment initiation	4.426 (4.005 to 4.848)	**<0.001**
Height	Male sex	2.126 (0.072 to 4.180)	**0.043**
Height	Initial GH dose	2.320 (0.947 to 3.692)	**<0.001**
Height	Pretreatment height SDS	3.624 (2.537 to 4.711)	**<0.001**
Weight	Year 2 vs Year 1	5.355 (3.685 to 7.025)	**<0.001**
Weight	Year 3 vs Year 1	10.824 (8.969 to 12.678)	**<0.001**
Weight	Year 4 vs Year 1	14.654 (12.420 to 16.887)	**<0.001**
Weight	Year 5 vs Year 1	15.840 (13.307 to 18.372)	**<0.001**
Weight	GHD vs ISS	0.052 (−2.205 to 2.309)	0.964
Weight	Year 2 × GHD	−0.525 (−2.675 to 1.624)	0.632
Weight	Year 3 × GHD	−1.350 (−3.754 to 1.054)	0.271
Weight	Year 4 × GHD	−2.858 (−5.779 to 0.063)	0.055
Weight	Year 5 × GHD	−2.275 (−5.791 to 1.241)	0.205
Weight	Age at treatment initiation	1.493 (1.075 to 1.910)	**<0.001**
Weight	Male sex	1.920 (−0.109 to 3.949)	0.064
Weight	Initial GH dose	8.903 (7.535 to 10.270)	**<0.001**
Weight	Pretreatment height SDS	0.142 (−0.925 to 1.210)	0.794
Annual height gain	Year 2 vs Year 1	−0.694 (−2.105 to 0.717)	0.335
Annual height gain	Year 3 vs Year 1	−1.337 (−2.870 to 0.196)	0.087
Annual height gain	Year 4 vs Year 1	−3.375 (−5.187 to −1.563)	**<0.001**
Annual height gain	Year 5 vs Year 1	−4.622 (−6.673 to −2.570)	**<0.001**
Annual height gain	GHD vs ISS	0.291 (−0.976 to 1.558)	0.653
Annual height gain	Year 2 × GHD	−0.545 (−2.362 to 1.272)	0.557
Annual height gain	Year 3 × GHD	−1.141 (−3.143 to 0.861)	0.264
Annual height gain	Year 4 × GHD	−0.187 (−2.569 to 2.195)	0.878

Outcome	Model term	Estimate (95% CI) or test	P value
Annual height gain	Year 5 × GHD	−0.274 (−3.160 to 2.612)	0.852
Annual height gain	Age at treatment initiation	−0.111 (−0.279 to 0.058)	0.198
Annual height gain	Male sex	0.929 (0.143 to 1.716)	**0.021**
Annual height gain	Initial GH dose	0.175 (−0.406 to 0.757)	0.555
Annual height gain	Pretreatment height SDS	−0.709 (−1.098 to −0.320)	**<0.001**
Annual weight gain	Year 2 vs Year 1	0.923 (−0.688 to 2.533)	0.261
Annual weight gain	Year 3 vs Year 1	1.266 (−0.483 to 3.015)	0.156
Annual weight gain	Year 4 vs Year 1	0.975 (−1.093 to 3.043)	0.355
Annual weight gain	Year 5 vs Year 1	−1.476 (−3.817 to 0.865)	0.216
Annual weight gain	GHD vs ISS	0.484 (−0.955 to 1.924)	0.510
Annual weight gain	Year 2 × GHD	−0.990 (−3.059 to 1.079)	0.348
Annual weight gain	Year 3 × GHD	−1.161 (−3.441 to 1.118)	0.318
Annual weight gain	Year 4 × GHD	−2.190 (−4.904 to 0.524)	0.114
Annual weight gain	Year 5 × GHD	0.941 (−2.349 to 4.231)	0.575
Annual weight gain	Age at treatment initiation	0.297 (0.105 to 0.488)	**0.002**
Annual weight gain	Male sex	0.696 (−0.200 to 1.591)	0.128
Annual weight gain	Initial GH dose	0.602 (−0.062 to 1.265)	0.075
Annual weight gain	Pretreatment height SDS	−0.300 (−0.744 to 0.145)	0.186
Height	Overall time	Overall test	**<0.001**
Height	Overall group	Overall test	0.228
Height	Time × group	Overall test	0.941
Weight	Overall time	Overall test	**<0.001**
Weight	Overall group	Overall test	0.964
Weight	Time × group	Overall test	0.340
Annual height gain	Overall time	Overall test	**<0.001**
Annual height gain	Overall group	Overall test	0.653
Annual height gain	Time × group	Overall test	0.853
Annual weight gain	Overall time	Overall test	0.169
Annual weight gain	Overall group	Overall test	0.510
Annual weight gain	Time × group	Overall test	0.392

Note. *Estimates are unstandardized fixed-effect coefficients with 95% confidence intervals from adjusted linear mixed-effects models. Models were adjusted for age at treatment initiation, sex, initial GH dose, and pretreatment height SDS. Year 1 and the ISS group were the reference categories; therefore, "GHD vs ISS" represents the group difference at Year 1, and the year-by-GHD terms represent differences in change from Year 1 between groups. Overall time, group, and time-by-group p values are omnibus tests. Units are cm for height, kg for weight, cm/year for annual height gain, and kg/year for annual weight gain. Singular fits were reported for the annual growth-gain models; interpret those estimates cautiously.*

Abbreviations: *CI, confidence interval; GHD, growth hormone deficiency; GH, growth hormone; ISS, idiopathic short stature; SDS, standard deviation score.*

Supplementary Table S5. Baseline Characteristics by Follow-Up Completeness

Characteristic	Complete follow-up	Incomplete follow-up	P value
**Age at treatment initiation, years ***	6.87 ± 2.55 (n=16)	12.58 (10.04–14.16) (n=58)	**<0.001**
**Sex, male**	10/16 (62.5%)	42/58 (72.4%)	0.442
**Treatment group, ISS**	8/16 (50.0%)	20/58 (34.5%)	0.257
**Treatment group, GHD**	8/16 (50.0%)	38/58 (65.5%)	
**Pretreatment height, cm***	101.14 ± 17.22 (n=16)	132.00 (119.70–143.80) (n=57)	**<0.001**
**Pretreatment height SDS***	−3.20 (−4.05 to −2.79) (n=16)	−2.55 (−2.82 to −2.50) (n=56)	**<0.001**
**Pretreatment weight, kg***	16.02 ± 6.04 (n=16)	29.00 (21.70–38.00) (n=57)	**<0.001**
**Baseline IGF-1 (μg/L)***	89.19 ± 56.19 (n=16)	148.50 (82.47–229.00) (n=56)	**0.008**
**Initial GH dose (mg/kg/day)***	0.03 (0.03–0.04) (n=16)	0.03 (0.03–0.03) (n=57)	0.105
**Pituitary MRI, normal**	14/16 (87.5%)	49/57 (86.0%)	>0.999

Note. *Complete follow-up was defined as non-missing height and weight measurements at all five annual time points. Categorical variables are presented as n (%). Continuous variables are reported as mean ± SD for approximately normally distributed data and as median (IQR) for skewed data.*

Abbreviations: *GHD, growth hormone deficiency; GH, growth hormone; IGF-1, insulin-like growth factor 1; ISS, idiopathic short stature; MRI, magnetic resonance imaging; SDS, standard deviation score.*

Supplementary Table S6. Frequency, Genomic Location, Zygosity, and ACMG/AMP Classification of GH1 Sequence Variants

Variant (dbSNP ID/position)	Genomic location	Nucleotide change (HGVS)	Amino acid change	Functional consequence	Carriers, n (%)	Het, n	Hom, n	ACMG/AMP class
rs2005172	Promoter/upstream				67 (90.5)	34	33	Not classified
rs6171	Promoter/upstream				52 (70.3)	43	9	Likely benign
rs2005171	Promoter/upstream				38 (51.4)	30	8	Benign
rs1811081	Promoter/upstream				22 (29.7)	19	3	Benign
rs2011732	Promoter/upstream				22 (29.7)	19	3	Not classified
rs11568828	Promoter/upstream				13 (17.6)	11	2	Benign
rs11568827	Promoter/upstream				12 (16.2)	12	0	Benign
rs2727338	Promoter/upstream				2 (2.7)	2	0	Not classified
rs71651678	Promoter/upstream				2 (2.7)	2	0	Not classified
rs71651679	Promoter/upstream				2 (2.7)	2	0	Likely benign
rs61762493	Promoter/upstream				1 (1.4)	1	0	Not classified
rs68096070	Promoter/upstream				1 (1.4)	1	0	Not classified
rs71651675	Promoter/upstream				1 (1.4)	1	0	Not classified
rs695	5' UTR				10 (13.5)	10	0	Likely benign
rs6173	5' UTR				6 (8.1)	6	0	Likely benign
rs6175	5' UTR				3 (4.1)	3	0	Not classified
rs6172	5' UTR				2 (2.7)	2	0	Likely benign
rs9282699	5' UTR				2 (2.7)	2	0	Likely benign
rs2665802	Intronic				50 (67.6)	36	14	Likely benign
rs3744287	Intronic				4 (5.4)	4	0	Benign
rs41295245	Intronic				3 (4.1)	3	0	Benign
rs41295031	Intronic				2 (2.7)	2	0	Not classified
chr17:61994885 G>C	Intronic				1 (1.4)	1	0	Not classified

Variant (dbSNP ID/position)	Genomic location	Nucleotide change (HGVS)	Amino acid change	Functional consequence	Carriers, n (%)	Het, n	Hom, n	ACMG/AMP class
rs199749063	Intronic				1 (1.4)	1	0	Not classified
rs560952118	Intronic				1 (1.4)	1	0	Not classified
rs71640281	Intronic				1 (1.4)	1	0	Not classified
rs976368253	Intronic				1 (1.4)	1	0	Likely benign
rs2001345	Exonic	NM_022559.3:c.7A>G	p.T3A	nonsynonymous SNV	3 (4.1)	3	0	Likely benign
chr17:61994742 T>C	Exonic	NM_022559.4:c.536A>G	p.K179R	nonsynonymous SNV	1 (1.4)	1	0	Not classified
chr17:61995733 C>G	Exonic	NM_022560.4:c.144G>C	p.Q48H	nonsynonymous SNV	1 (1.4)	1	0	Not classified
chr17:61995763 G>-	Exonic	NM_022560.4:c.114delC	p.N38fs	frameshift deletion	1 (1.4)	1	0	Not classified
rs150761983	Exonic	NM_022559.4:c.283C>G	p.Q95E	nonsynonymous SNV	1 (1.4)	1	0	Uncertain significance
chr17:61994283 C>T	Downstream				1 (1.4)	1	0	Not classified
chr17:61994287 C>T	Downstream				1 (1.4)	1	0	Not classified
chr17:61994291 T>C	Downstream				1 (1.4)	1	0	Not classified
chr17:61994354 G>T	Downstream				1 (1.4)	1	0	Not classified
chr17:61994362 A>T	Downstream				1 (1.4)	1	0	Not classified
chr17:61994364 A>T	Downstream				1 (1.4)	1	0	Not classified
chr17:61994398 C>A	Downstream				1 (1.4)	1	0	Not classified
chr17:61994400 C>T	Downstream				1 (1.4)	1	0	Not classified
chr17:61994401 A>G	Downstream				1 (1.4)	1	0	Not classified
chr17:61994412 A>G	Downstream				1 (1.4)	1	0	Not classified
rs111508178	Downstream				1 (1.4)	1	0	Likely benign
rs60590949	Downstream				1 (1.4)	1	0	Likely benign
rs933006846	Downstream				1 (1.4)	1	0	Likely benign

Note. *Variants are listed in the GRCh37/hg19 reference context. Carrier percentages are based on the total cohort (N = 74). "Carriers" indicates the number of participants with each variant; "Het" and "Hom" indicate heterozygous and homozygous carriers, respectively. Participants may carry multiple variants, so counts across rows and genomic regions are not mutually exclusive. ACMG/AMP classifications are shown as reported; "Not classified" indicates that no classification was assigned. Variants without a dbSNP identifier are reported by genomic coordinate and reference-to-alternate allele.*

Abbreviations: *ACMG/AMP, American College of Medical Genetics and Genomics/Association for Molecular Pathology; Het, heterozygous; Hom, homozygous; HGVS, Human Genome Variation Society; SNV, single-nucleotide variant; UTR, untranslated region.*

Supplementary Table S7. Descriptive Summary of Exploratory GH1 Variant Clustering Analyses

Analysis	Parameter	Result
MCA + k-means clustering	Number of MCA dimensions	5
MCA + k-means clustering	Optimal number of clusters	3
MCA + k-means clustering	Average silhouette coefficient	0.681
MCA + k-means clustering	Cluster 1	71 (95.9%)
MCA + k-means clustering	Cluster 2	2 (2.7%)
MCA + k-means clustering	Cluster 3	1 (1.4%)
Jaccard + hierarchical clustering	Optimal number of clusters	3
Jaccard + hierarchical clustering	Average silhouette coefficient	0.306
Jaccard + hierarchical clustering	Cluster 1	45 (60.8%)
Jaccard + hierarchical clustering	Cluster 2	3 (4.1%)
Jaccard + hierarchical clustering	Cluster 3	22 (29.7%)
Jaccard + hierarchical clustering	Empty (0 variants)	4 (5.4%)

Note. *Clustering analyses were exploratory and descriptive. Percentages are based on the full cohort (N = 74). The MCA-based k-means solution used five MCA dimensions and assigned 71 participants to one cluster, indicating a highly imbalanced cluster distribution. In the Jaccard similarity–based hierarchical analysis, "Empty (0 variants)" denotes the four participants with no detected GH1 variants; they were not assigned to a cluster. Average silhouette coefficients are reported for each method as a descriptive measure of cluster separation. Given the exploratory design and imbalanced cluster sizes, the results should not be interpreted as evidence of distinct biological subgroups.*

Abbreviations: *GH1, growth hormone 1; MCA, multiple correspondence analysis.*

Supplementary Figures

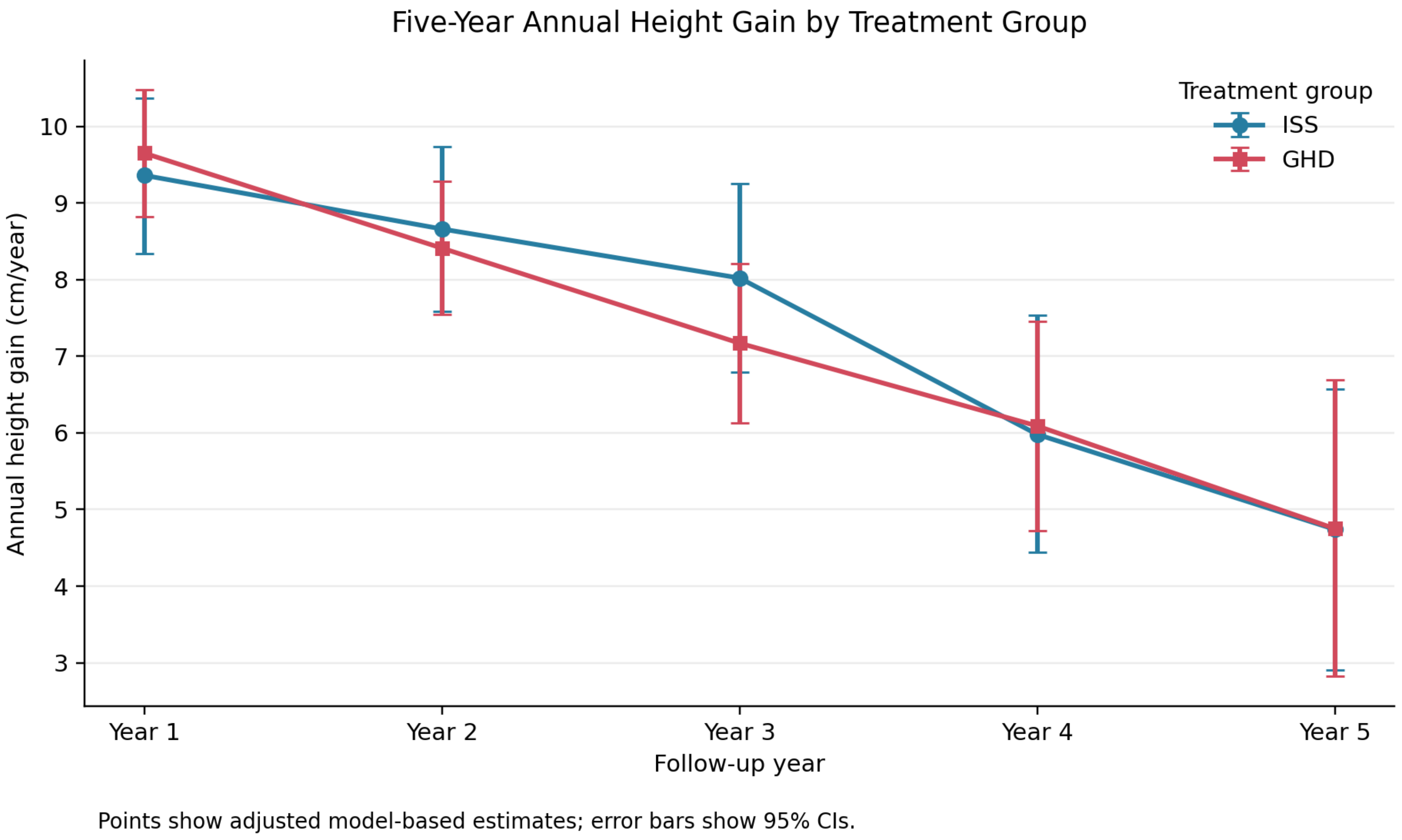

Supplementary Figure S1. Five-Year Annual Height Gain by Treatment Group

The x-axis shows follow-up year, and the y-axis shows annual height gain (cm/year). Points show adjusted estimates from the linear mixed-effects model; error bars indicate 95% CIs. Available observations (ISS/GHD) at Years 1–5 were 27/45, 24/36, 18/25, 11/15, and 8/7, respectively. The model was adjusted for age at treatment initiation, sex, initial GH dose, and pretreatment height SDS. There was an overall time effect ($p<0.001$), but no significant treatment-group effect ($p=0.653$) or time-by-group interaction ($p=0.853$).

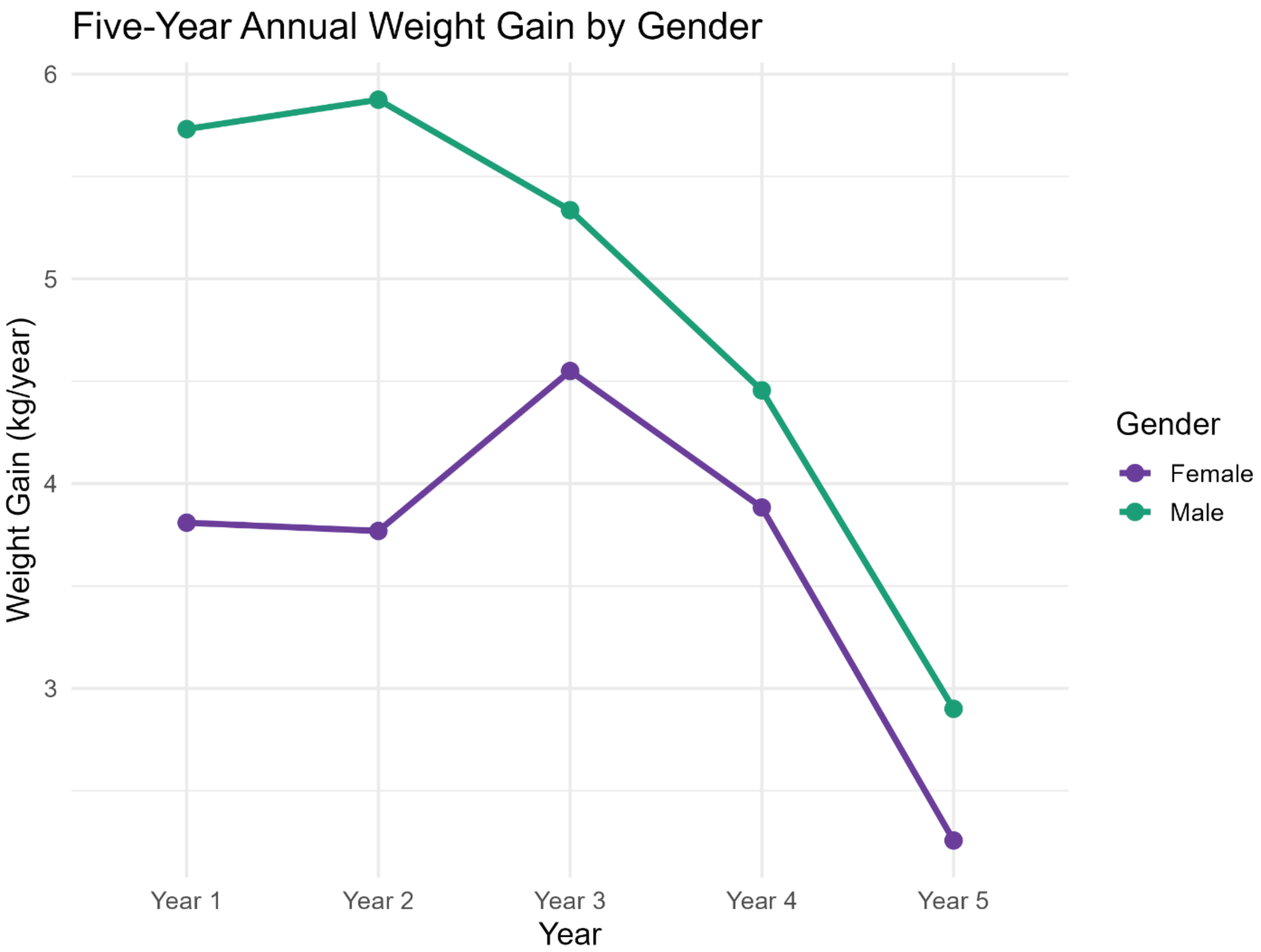

Supplementary Figure S2. Five-Year Annual Weight Gain by Gender

The x-axis shows follow-up year, and the y-axis shows weight (kg). Lines show adjusted estimates from the linear mixed-effects model; error bars indicate 95% confidence intervals. Available observations (ISS/GHD) were: Year 1, 27/46; Year 2, 24/37; Year 3, 18/27; Year 4, 11/16; and Year 5, 8/8. The model was adjusted for age at treatment initiation, sex, initial GH dose, and pretreatment height SDS. There was an overall time effect ($p<0.001$), but no significant treatment-group effect ($p=0.964$) or time-by-group interaction ($p=0.340$).

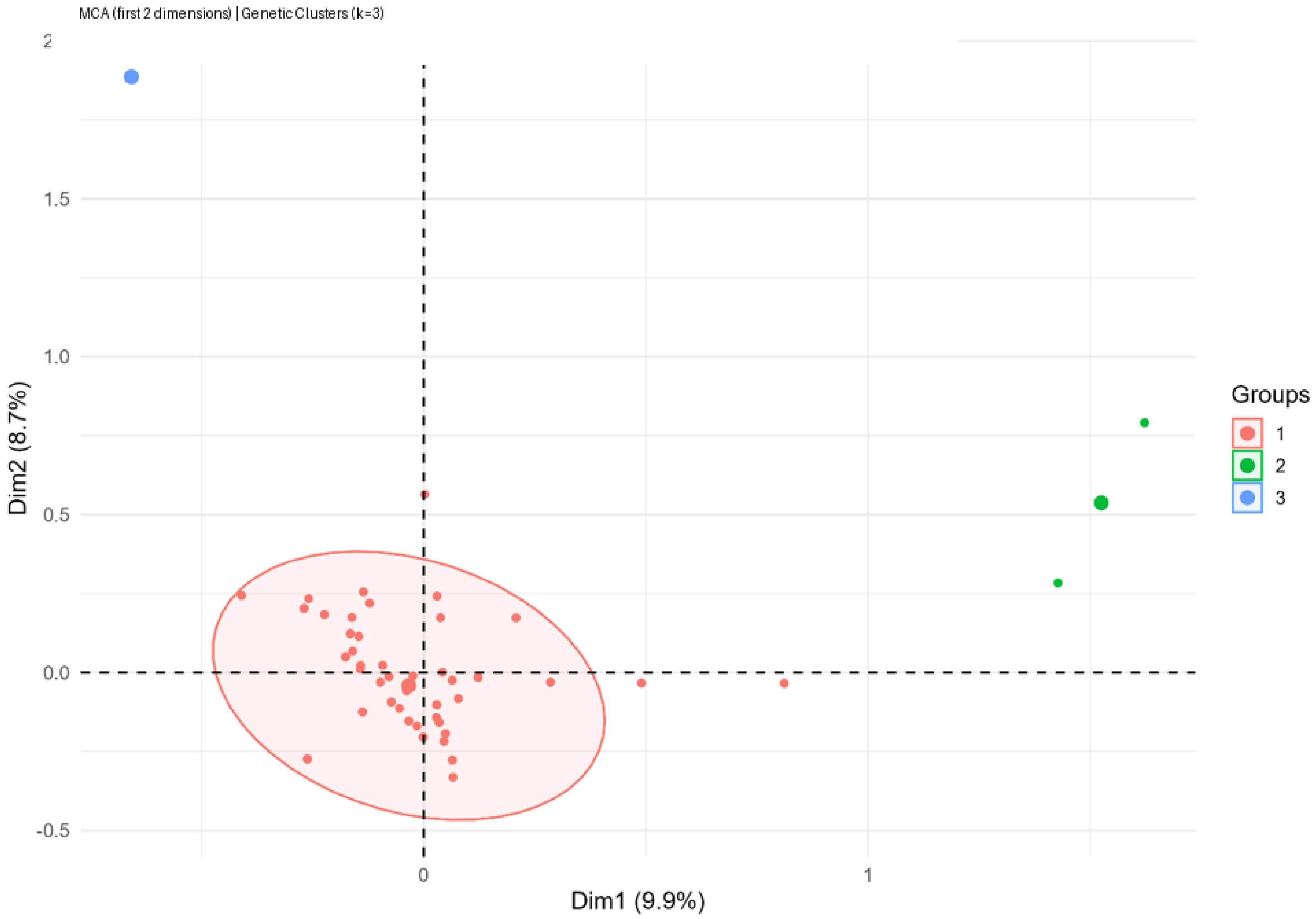

Supplementary Figure S3. MCA-Based Visualization of GH1 Variant Clusters

The first two multiple correspondence analysis dimensions are shown (Dimension 1, 9.9% of inertia; Dimension 2, 8.7%). Points are colored by the three clusters from the MCA-based k-means solution. Cluster sizes were 71, 2, and 1 participants. This visualization is exploratory; the highly imbalanced cluster distribution limits interpretation as distinct biological subgroups.
